# Supplementary material for: Induction of Acquired Resistance towards EGFR Inhibitor Gefitinib in a Patient-Derived Xenograft Model of Non-Small Cell Lung Cancer and Subsequent Molecular Characterization
Source: Cells. 2019 Jul 18;8(7):740. doi: 10.3390/cells8070740 (PMC6678194; doi:10.3390/cells8070740)
Supplement: Supplementary file 1 [file cells-08-00740-s001.pdf]

cells

Article

Supplementary Materials: The following are available online at www.mdpi.com/xxx/s1,

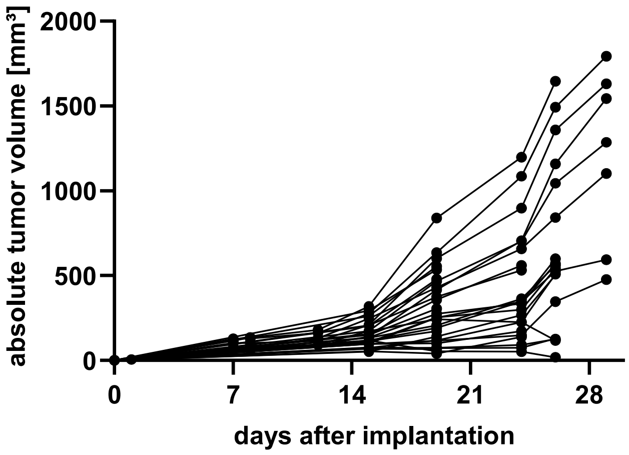

Supplemental Figure S1. Tumor growth behavior of subcutaneously implanted LXFA 677 in vivo. LXFA 677 was implanted subcutaneously into NMRI nude mice. Tumor volume was measured twice weekly from the day of implantation for four weeks.

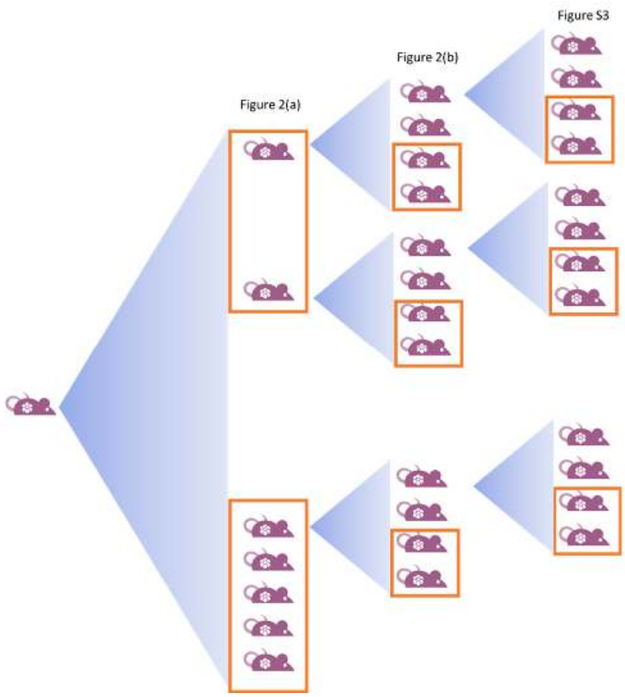

Supplemental Figure S2: Induction of resistance in NSCLC PDX LXFA 677 in vivo. Flowchart of the transplantation and treatment process of LXFA 677 and the three resistant sublines. Untreated LXFA 677 PDX material was transplanted subcutaneously into six NMRI nude mice and Gefitinib treatment was performed as

described in Figure 2 (a). Three tumor sublines, called LXFA 677res1, -res2 and -res3, showed progressive growth under constant treatment with Gefitinib and were re-transplanted into four NMRI nude mice, respectively. The four mice were stratified into a control group receiving the vehicle or the treatment group receiving Gefitinib as described in Figure 2 (b). One untreated control tumor of each subline served as donor material for a subsequent implantation into four NMRI nude mice. Again animals were stratified into control and treatment arm and Gefitinib was dosed as described in supplemental Figure S5. The orange box is indicating Gefitinib treatment

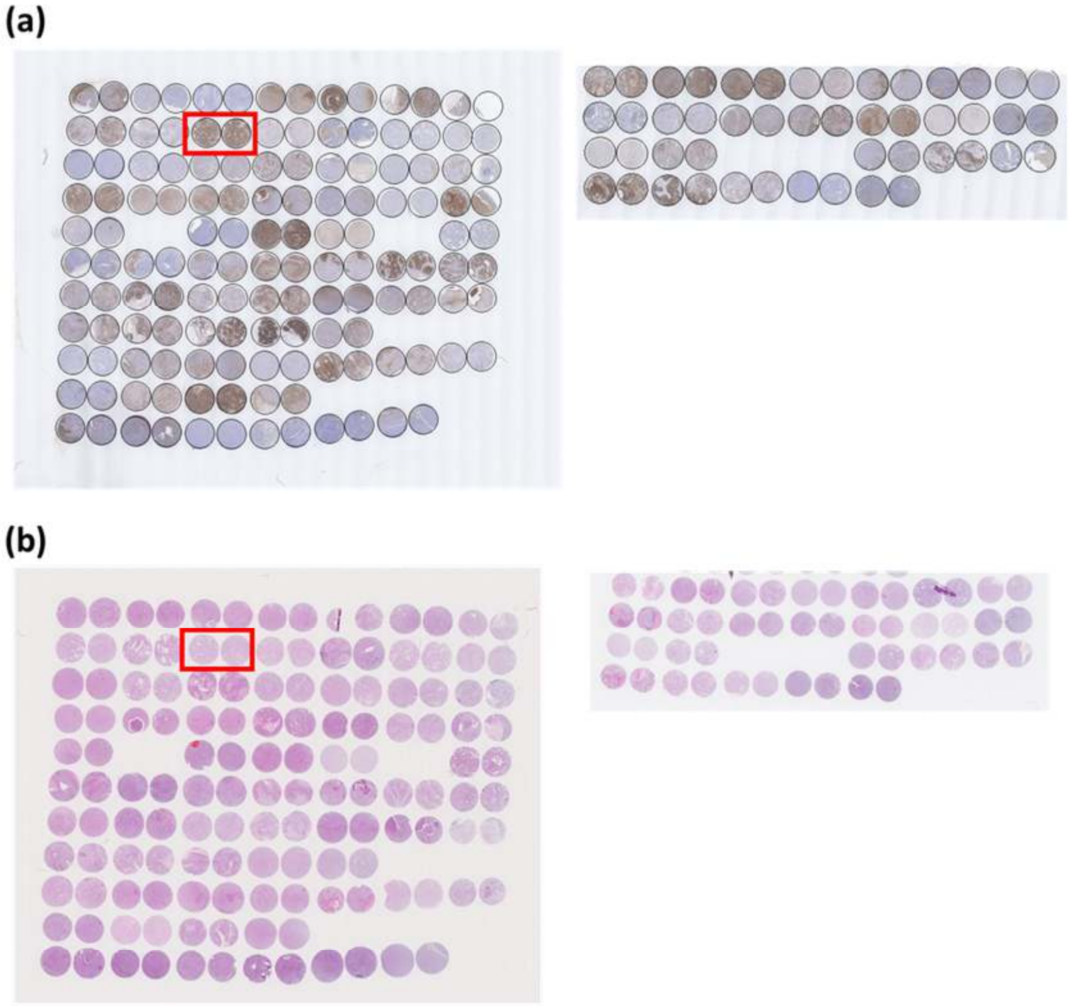

(c)

LXFA 289	LXFA 297	LXFA 400	LXFA 526	LXFA 586	LXFA 592	LXFA 623
LXFA 629	LXFA 644	LXFA 677	LXFA 737	LXFA 749	LXFA 923	LXFA 983
LXFA 1012	LXFA 1041	LXFA 1335	LXFA 1584	LXFA 1647	LXFA 1848	LXFA 2155
LXFA 2158	LXFA 2165	LXFA 2184	LXFA 2201	LXFA 2204	LXFA 2207	LXFA 2217
LXFA 2250		LXF 1125	LXFE 2478	LXFA 2764		LXA 3109
LXFE 211	LXFE 397	LXFE 409	LXFE 470	LXFE 597	LXFE 646	LXFE 690
LXFE 772	LXFE 937	LXFE 1022	LXFE 1066	LXFE 1422	LXFE 2159	LXFE 2162
LXFE 2226	LXFE 2232	LXFE 2257	LXFE 2348	LXFE 2415		
LXFL 430	LXFL 529	LXFL 625	LXFL 1072	LXFL 1121	LXFL 1176	LXFL 1674
LXFL 2228	LXFL 2237	LXFE 2324	LXFL 2377			
LXFS 538	LXFS 573	LXFS 615	LXFS 650	LXFS 1129	LXFS 2156	

LXA 3100	LXA 3104	LXA 3106	LXAA SMTCA62	LXF 2436	LXF 2467	LXFA 2157
LXFA 2160	LXFA 2194	LXFA 2366	LXFA 2388	LXFA 2403	LXFA 2425	LXFA 2426
LXFA 2764	LXFA 2889			LXFE 386	LXFE 2190	LXFE 2198
LXFE 2214	LXFE 2220	LXFE 2276	LXFS 605	LXFS 688		

Figure S3: TMA of the NSCLC PDX panel comprising 85 NSCLC PDX models in duplicates. The red box is indicating tumor model LXFA 677. (a): IHC with anti-human EGFR Antibody followed by DAB staining and hematoxylin counterstaining (b): Hematoxylin& Eosin staining (c): Mapping of the individual tumor models on the TMA. The red box is indicating LXFA 677 model.

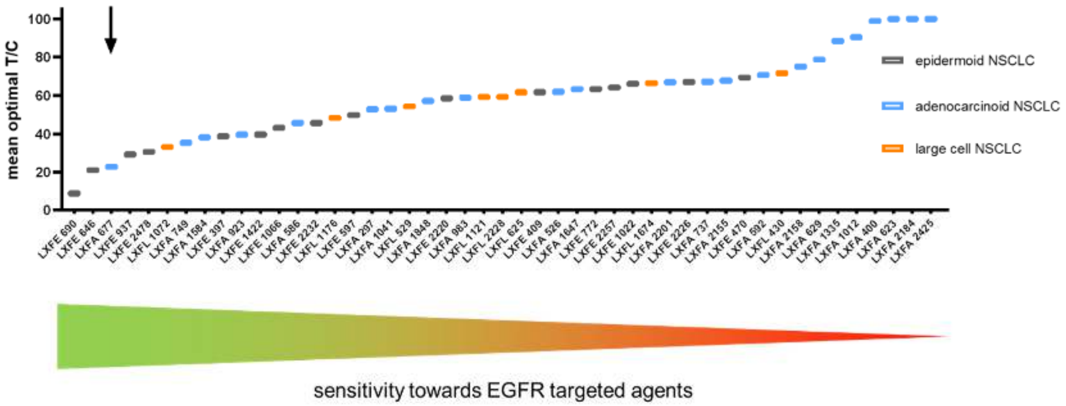

Supplemental Figure S4: Sensitivity towards EGFR targeting agents of 47 NSCLC PDX *in vivo*. Depicted are the mean optimal T/C values of one – five EGFR targeting agents per model. In total nine different compounds were tested: Osimertinib, Afatinib, Cetuximab, Erlotinib, Gefitinib, Necitumumab, Lapatinib, Sorafenib and Sunitinib. Dosing and schedule are depicted in Table 2. The respective tumor model was implanted subcutaneously in NMRI nude mice and treatment started when a group median tumor volume of 250 mm³ was reached. The group size was n = 5 -10 animals/group.

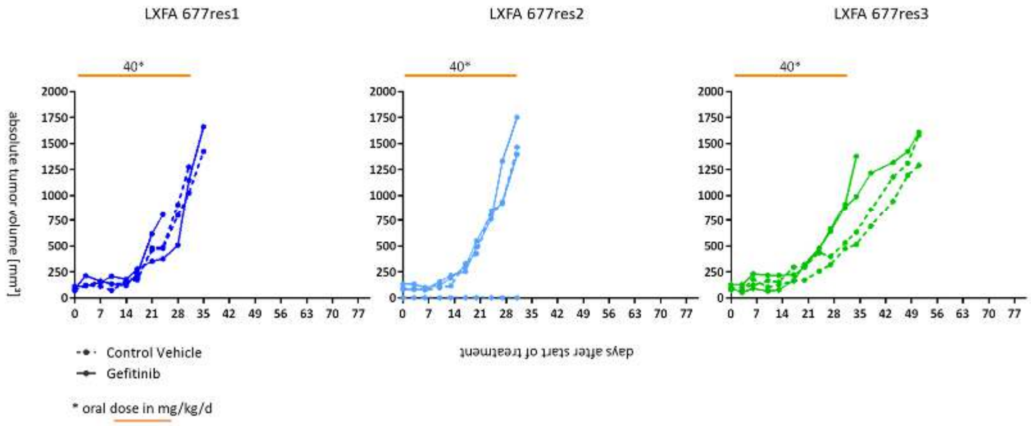

Figure S5. The orange box is indicating the mice receiving Gefitinib treatment: Tumor growth curves for individual tumors after re-implantation of tumors from the untreated control groups of the resistant lines LXFA 677res1, -res2 and res3. The orange line is indicating the duration of the treatment. The respective dose per day is shown above the line.

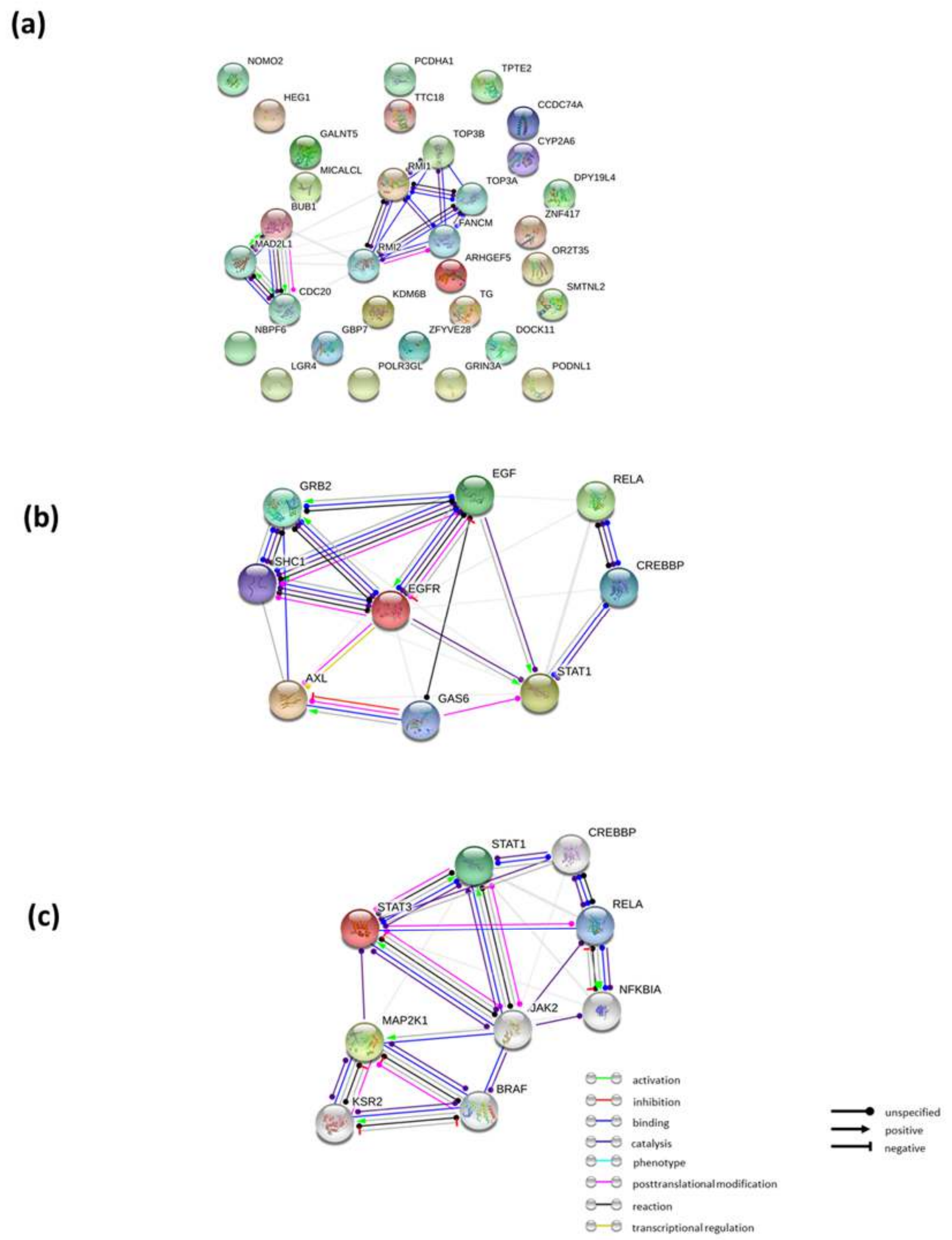

Figure S6: Functional protein association networks of (a): Genes found to be mutated in all three Gefitinib-resistant LXFA 677 subclones (b): Functional protein association networks of proteins with an upregulated logFC > 0.5 in the resistant lines (c): Functional protein association networks of proteins with an upregulated logFC > 0.5 in the phosphorylated vs total protein ratio. The STRING (https://string-db.org/) program was used to assign mutated genes and the upregulated proteins to a protein network. As a minimum required interaction score the medium confidence level was applied (p= 0.400) allowing a maximal number of five interactors. Analysis of the involvement of the mutated genes/upregulated proteins in biological processes and functions is based on GO-term and KEGG pathways.

Table S1. Number of common and unique mutations in the different sublines of NSCLC PDX LXFA 677

	LXFA 677	LXFA 677res1	LXFA 677res2	LXFA 677res3
common	504	504	504	504
in 2/3 sublines	26	30	42	38
unique	46	53	28	26
total	576	587	574	568

Table S2. Annotation of mutations shared among all three resistant lines based on SIFT and PolyPhen. nd = dot determined

**SYMBOL**	**Transcript**	**HGVSg Nomenclature**	**Consequence (ENSEMBL)**	**Amino_acid_change**	**SIFT prediction**	**PolyPhen prediciton**	**Mutation existent in LXFE_2478**
GBP7	ENST00000294671	1:g.89133401G>C	missense_variant	Q507E	tolerated	benign	No
NBPF6	ENST00000495380	1:g.108459067G>A	missense_variant	S329N	tolerated	benign	No
OR2T35	ENST00000641268	1:g.248639168C>T	missense_variant	V31I	tolerated	benign	Yes
CCDC74A	ENST00000295171	2:g.131532868G>A	missense_variant	R294Q	tolerated	benign	No
HEG1	ENST00000311127	3:g.125013588_125013605dup	inframe_insertion	S668SSSSSSS	nd	nd	Yes
ARHGEF5	ENST00000056217	7:g.144374813C>A	missense_variant	L1408I	deleterious	benign	No
MICALCL	ENST00000256186	11:g.12294836_12294841dup	inframe_insertion	A456APP	nd	nd	Yes
TPTE2	ENST00000400230	13:g.19492854T>C	missense_variant	K39E	tolerated - low confidence	benign	Yes
TPTE2	ENST00000400230	13:g.19492871A>C	missense_variant	L33R	tolerated - low confidence	benign	Yes
NOMO2	ENST00000621364	16:g.18531526C>T	missense_variant	V493M	deleterious	possibly damaging	No
SMTNL2	ENST00000389313	17:g.4592373G>A	missense_variant	D138N	deleterious	benign	No
KDM6B	ENST00000254846	17:g.7846893_7846898dup	inframe_insertion	L251LPP	nd	nd	Yes
ZNF417	ENST00000312026	19:g.57909712G>T	missense_variant	A189E	tolerated	benign	No
DOCK11	ENST00000276202	X:g.118598098A>C	missense_variant	E818D	nd	nd	Yes

Table S3. Most significant biological processes and functions (based on GO, KEGG and reactome pathway) in which the mutated genes in at least 2/3 of the resistant lines are involved.

**Pathway**	**#term ID**	**term description**	**observed gene count**	**background gene count**	**false discovery rate**	**matching proteins in our network**
GO	GO:0003917	DNA topoisomerase type I activity	2	4	0.0069	TOP3A,TOP3B
KEGG	hsa03460	Fanconi anemia pathway	5	51	9.34e-07	FANCM,RMI1,RMI2,TOP3A,TOP3B
	hsa04110	Cell cycle	3	123	0.0148	BUB1,CDC20,MAD2L1
	hsa04114	Oocyte meiosis	3	116	0.0148	BUB1,CDC20,MAD2L1
	hsa03440	Homologous recombination	2	40	0.0165	TOP3A,TOP3B
Reactome	HSA-69620	Cell Cycle Checkpoints	6	265	0.00049	BUB1,CDC20,MAD2L1,RMI1,RMI2,TOP3A
	HSA-73894	DNA Repair	4	290	0.0065	FANCM,RMI1,RMI2,TOP3A

Table S4. Most significant biological processes and functions in which the upregulated proteins are involved based on GO-term

**#term ID**	**term description**	**observed gene count**	**background gene count**	**false discovery rate**	**matching proteins in our network (labels)**
GO:0016032	viral process	8	571	6.59e-09	AXL, CREBBP, EGFR, GAS6, GRB2, RELA, SHC1, STAT1
GO:0038128	ERBB2 signaling pathway	4	31	3.60e-07	EGF, EGFR, GRB2, SHC1
GO:0007166	cell surface receptor signaling pathway	9	2198	7.80e-07	AXL, CREBBP, EGF, EGFR, GAS6, GRB2, RELA, SHC1, STAT1
GO:0051897	positive regulation of protein kinase B signaling	5	157	1.00e-06	AXL, EGF, EGFR, GAS6, GRB2
GO:0007173	epidermal growth factor receptor signaling pathway	4	52	1.44e-06	EGF, EGFR, GRB2, SHC1

Table S5. Most significant biological processes and functions in which the upregulated proteins are involved based on KEGG pathway.

**#term ID**	**term description**	**observed gene count**	**background gene count**	**false discovery rate**	**matching proteins in your network (labels)**
hsa01521	EGFR tyrosine kinase inhibitor resistance	6	78	4.80e-11	AXL, EGF, EGFR, GAS6, GRB2, SHC1
hsa05215	Prostate cancer	5	97	2.39e-08	CREBBP, EGF, EGFR, GRB2, RELA
hsa05160	Hepatitis C	5	131	5.75e-08	EGF, EGFR, GRB2, RELA, STAT1
hsa05165	Human papillomavirus infection	6	317	5.75e-08	CREBBP, EGF, EGFR, GRB2, RELA, STAT1
hsa04630	Jak-STAT signaling pathway	5	160	1.09e-07	CREBBP, EGF, EGFR, GRB2, STAT1
hsa04014	Ras signaling pathway	5	228	3.87e-07	EGF, EGFR, GRB2, RELA, SHC1
hsa04917	Prolactin signaling pathway	4	69	3.87e-07	GRB2, RELA, SHC1, STAT1
hsa05200	Pathways in cancer	6	515	3.87e-07	CREBBP, EGF, EGFR, GRB2, RELA, STAT1
hsa05212	Pancreatic cancer	4	74	3.87e-07	EGF, EGFR, RELA, STAT1
hsa05214	Glioma	4	68	3.87e-07	EGF, EGFR, GRB2, SHC1
hsa04012	ErbB signaling pathway	4	83	4.55e-07	EGF, EGFR, GRB2, SHC1
hsa04066	HIF-1 signaling pathway	4	98	7.94e-07	CREBBP, EGF, EGFR, RELA

Table S6. Most significant biological processes and functions in which the upregulated activated proteins are involved based on GO-term.

**#term ID**	**term description**	**observed gene count**	**background gene count**	**false discovery rate**	**matching proteins in your network (labels)**
GO:0035556	intracellular signal transduction	8	1528	3.96e-06	BRAF, JAK2, KSR2, MAP2K1, NFKBIA, RELA, STAT1, STAT3
GO:0042981	regulation of apoptotic process	8	1501	3.96e-06	BRAF, CREBBP, JAK2, MAP2K1, NFKBIA, RELA, STAT1, STAT3
GO:0070106	interleukin-27-mediated signaling pathway	3	11	4.03e-06	JAK2, STAT1, STAT3
GO:0070757	interleukin-35-mediated signaling pathway	3	11	4.03e-06	JAK2, STAT1, STAT3
GO:0009966	regulation of signal transduction	9	3033	4.73e-06	BRAF, CREBBP, JAK2, KSR2, MAP2K1, NFKBIA, RELA, STAT1, STAT3
GO:0071354	cellular response to interleukin-6	4	30	1.10e-06	JAK2, RELA, STAT1, STAT3
GO:0035556	intracellular signal transduction	8	1528	3.96e-06	BRAF, JAK2, KSR2, MAP2K1, NFKBIA, RELA, STAT1, STAT3
GO:0042981	regulation of apoptotic process	8	1501	3.96e-06	BRAF, CREBBP, JAK2, MAP2K1, NFKBIA, RELA, STAT1, STAT3
GO:0070106	interleukin-27-mediated signaling pathway	3	11	4.03e-06	JAK2, STAT1, STAT3

70

71 Table S7. Most significant biological processes and functions in which the upregulated activated proteins are involved based on KEGG pathway.

#term ID	term description	observed gene count	background gene count	false discovery rate	matching proteins in your network (labels)
hsa04062	Chemokine signaling pathway	7	181	2.84e-11	BRAF, JAK2, MAP2K1, NFKBIA, RELA, STAT1, STAT3
hsa05167	Kaposi's sarcoma-associated herpesvirus infection	7	183	2.84e-11	CREBBP, JAK2, MAP2K1, NFKBIA, RELA, STAT1, STAT3
hsa05200	Pathways in cancer	8	515	8.59e-11	BRAF, CREBBP, JAK2, MAP2K1, NFKBIA, RELA, STAT1, STAT3
hsa05161	Hepatitis B	6	142	4.14e-10	CREBBP, MAP2K1, NFKBIA, RELA, STAT1, STAT3
hsa05164	Influenza A	6	168	8.85e-10	CREBBP, JAK2, MAP2K1, NFKBIA, RELA, STAT1
hsa04917	Prolactin signaling pathway	5	69	1.66e-09	JAK2, MAP2K1, RELA, STAT1, STAT3
hsa05212	Pancreatic cancer	5	74	1.99e-09	BRAF, MAP2K1, RELA, STAT1, STAT3
hsa05215	Prostate cancer	5	97	6.41e-09	BRAF, CREBBP, MAP2K1, NFKBIA, RELA
hsa04659	Th17 cell differentiation	5	102	7.27e-09	JAK2, NFKBIA, RELA, STAT1, STAT3
hsa05145	Toxoplasmosis	5	109	9.03e-09	JAK2, NFKBIA, RELA, STAT1, STAT3
hsa05160	Hepatitis C	5	131	2.00e-08	BRAF, NFKBIA, RELA, STAT1, STAT3
hsa05162	Measles	5	133	2.00e-08	JAK2, NFKBIA, RELA, STAT1, STAT3
hsa05168	Herpes simplex infection	5	181	8.21e-08	CREBBP, JAK2, NFKBIA, RELA, STAT1
hsa04024	cAMP signaling pathway	5	195	1.10e-07	BRAF, CREBBP, MAP2K1, NFKBIA, RELA
hsa05221	Acute myeloid leukemia	4	66	1.48e-07	BRAF, MAP2K1, RELA, STAT3
hsa04920	Adipocytokine signaling pathway	4	69	1.64e-07	JAK2, NFKBIA, RELA, STAT3
hsa05140	Leishmaniasis	4	70	1.64e-07	JAK2, NFKBIA, RELA, STAT1
hsa05220	Chronic myeloid leukemia	4	76	2.12e-07	BRAF, MAP2K1, NFKBIA, RELA
hsa01521	EGFR tyrosine kinase inhibitor resistance	4	78	2.22e-07	BRAF, JAK2, MAP2K1, STAT3
hsa04658	Th1 and Th2 cell differentiation	4	88	3.36e-07	JAK2, NFKBIA, RELA, STAT1

72

Author Contributions: Conceptualization, J.S., C.T., H.K. and W.S.; methodology, C.T., K.K., L.K. A.-L. P. and E.O.; software, D.B. and H.K.; validation, J.S., E.O. L.K., H.K. and W.S.; formal analysis, J.S., L.K. and H.K.; investigation, C.T., K.K., E.O. and A.-L.P; data curation, C.T., K.K., A-L.P. and L.K.; writing—original draft preparation, J.S.; writing—review and editing, H.K., L.K. and W.S.; visualization, J.S. W.S. and H.K.; supervision, W.S.; project administration, J.S.; funding acquisition, J.S and W.S.

Funding: This work has been carried out as a part of the IMI-funded EU-consortium PREDECT (www.predect.eu).

Acknowledgments: We thank Anke Behnke, Stefanie Klingel, Volker Knauff, Dorothée Lenhardt, Bérengère Ouine, Yana Raeva and Sabine Rajkumar for excellent technical support.

Conflicts of Interest: The authors declare no conflict of interest.

References

1. Wang, Z., ErbB Receptors and Cancer. *Methods in molecular biology (Clifton, N.J.)* **2017,** *1652*, 3-35.
2. Antony, J.; Thiery, J. P.; Huang, R. Y., Epithelial-to-mesenchymal transition: Lessons from development, insights into cancer and the potential of EMT-subtype based therapeutic intervention. *Physical biology* **2019**.
3. Siu, M. K.; Abou-Kheir, W.; Yin, J. J.; Chang, Y. S.; Barrett, B.; Suau, F.; Casey, O.; Chen, W. Y.; Fang, L.; Hynes, P.; Hsieh, Y. Y.; Liu, Y. N.; Huang, J.; Kelly, K., Loss of EGFR signaling regulated miR-203 promotes prostate cancer bone metastasis and tyrosine kinase inhibitors resistance. *Oncotarget* **2014,** *5* (11), 3770-84.
4. Tulchinsky, E.; Demidov, O.; Kriajevska, M.; Barlev, N. A.; Imyanitov, E., EMT: A mechanism for escape from EGFR-targeted therapy in lung cancer. *Biochimica et biophysica acta. Reviews on cancer* **2019,** *1871* (1), 29-39.
5. Xu, J.; Wang, J.; Zhang, S., Mechanisms of resistance to irreversible epidermal growth factor receptor tyrosine kinase inhibitors and therapeutic strategies in non-small cell lung cancer. *Oncotarget* **2017,** *8* (52), 90557-90578.
6. Zhu, X.; Bao, Y.; Guo, Y.; Yang, W., Proline-Rich Protein Tyrosine Kinase 2 in Inflammation and Cancer. *Cancers* **2018,** *10* (5).
7. Takeda, M.; Nakagawa, K., First- and Second-Generation EGFR-TKIs Are All Replaced to Osimertinib in Chemo-Naive EGFR Mutation-Positive Non-Small Cell Lung Cancer? *International journal of molecular sciences* **2019,** *20* (1), 146.
8. Kobayashi, S.; Boggon, T. J.; Dayaram, T.; Jänne, P. A.; Kocher, O.; Meyerson, M.; Johnson, B. E.; Eck, M. J.; Tenen, D. G.; Halmos, B., EGFR Mutation and Resistance of Non–Small-Cell Lung Cancer to Gefitinib. *New England Journal of Medicine* **2005,** *352* (8), 786-792.
9. Ohashi, K.; Maruvka, Y. E.; Michor, F.; Pao, W., Epidermal Growth Factor Receptor Tyrosine Kinase Inhibitor–Resistant Disease. *Journal of Clinical Oncology* **2013,** *31* (8), 1070-1080.
10. Sequist, L. V.; Waltman, B. A.; Dias-Santagata, D.; Digumarthy, S.; Turke, A. B.; Fidias, P.; Bergethon, K.; Shaw, A. T.; Gettinger, S.; Cosper, A. K.; Akhavanfard, S.; Heist, R. S.; Temel, J.; Christensen, J. G.; Wain, J. C.; Lynch, T. J.; Vernovsky, K.; Mark, E. J.; Lanuti, M.; Iafrate, A. J.; Mino-Kenudson, M.; Engelman, J. A., Genotypic and Histological Evolution of Lung Cancers Acquiring Resistance to EGFR Inhibitors. *Science Translational Medicine* **2011,** *3* (75), 75ra26-75ra26.
11. Herter-Sprie, G. S.; Kung, A. L.; Wong, K. K., New cast for a new era: preclinical cancer drug development revisited. *The Journal of clinical investigation* **2013,** *123* (9), 3639-45.

12. Hidalgo, M.; Amant, F.; Biankin, A. V.; Budinska, E.; Byrne, A. T.; Caldas, C.; Clarke, R. B.; de Jong, S.; Jonkers, J.; Maelandsmo, G. M.; Roman-Roman, S.; Seoane, J.; Trusolino, L.; Villanueva, A., Patient-derived xenograft models: an emerging platform for translational cancer research. *Cancer discovery* **2014,** *4* (9), 998-1013.
13. Teicher, B. A., Tumor models for efficacy determination. *Molecular cancer therapeutics* **2006,** *5* (10), 2435-43.
14. Tentler, J. J.; Tan, A. C.; Weekes, C. D.; Jimeno, A.; Leong, S.; Pitts, T. M.; Arcaroli, J. J.; Messersmith, W. A.; Eckhardt, S. G., Patient-derived tumour xenografts as models for oncology drug development. *Nature reviews. Clinical oncology* **2012,** *9* (6), 338-50.
15. Conway, T.; Wazny, J.; Bromage, A.; Tymms, M.; Sooraj, D.; Williams, E. D.; Beresford-Smith, B., Xenome—a tool for classifying reads from xenograft samples. *Bioinformatics* **2012,** *28* (12), i172-i178.
16. McLaren, W.; Gil, L.; Hunt, S. E.; Riat, H. S.; Ritchie, G. R. S.; Thormann, A.; Flicek, P.; Cunningham, F., The Ensembl Variant Effect Predictor. *Genome Biology* **2016,** *17* (1), 122.
17. Sherry, S. T.; Ward, M. H.; Kholodov, M.; Baker, J.; Phan, L.; Smigielski, E. M.; Sirotkin, K., dbSNP: the NCBI database of genetic variation. *Nucleic acids research* **2001,** *29* (1), 308-11.
18. Dayem Ullah, A. Z.; Oscanoa, J.; Wang, J.; Nagano, A.; Lemoine, N. R.; Chelala, C., SNPnexus: assessing the functional relevance of genetic variation to facilitate the promise of precision medicine. *Nucleic acids research* **2018,** *46* (W1), W109-W113.
19. Meseure, D.; Vacher, S.; Lallemand, F.; Alsibai, K. D.; Hatem, R.; Chemlali, W.; Nicolas, A.; De Koning, L.; Pasmant, E.; Callens, C.; Lidereau, R.; Morillon, A.; Bieche, I., Prognostic value of a newly identified MALAT1 alternatively spliced transcript in breast cancer. *British journal of cancer* **2016,** *114* (12), 1395-404.
20. Troncale, S.; Barbet, A.; Coulibaly, L.; Henry, E.; He, B.; Barillot, E.; Dubois, T.; Hupe, P.; de Koning, L., NormaCurve: a SuperCurve-based method that simultaneously quantifies and normalizes reverse phase protein array data. *PloS one* **2012,** *7* (6), e38686.
21. Szklarczyk, D.; Gable, A. L.; Lyon, D.; Junge, A.; Wyder, S.; Huerta-Cepas, J.; Simonovic, M.; Doncheva, N. T.; Morris, J. H.; Bork, P.; Jensen, L. J.; Mering, C. V., STRING v11: protein-protein association networks with increased coverage, supporting functional discovery in genome-wide experimental datasets. *Nucleic acids research* **2019,** *47* (D1), D607-d613.
22. Ritchie, M. E.; Phipson, B.; Wu, D.; Hu, Y.; Law, C. W.; Shi, W.; Smyth, G. K., limma powers differential expression analyses for RNA-sequencing and microarray studies. *Nucleic acids research* **2015,** *43* (7), e47-e47.
23. Morgillo, F.; Della Corte, C. M.; Fasano, M.; Ciardiello, F., Mechanisms of resistance to EGFR-targeted drugs: lung cancer. *ESMO open* **2016,** *1* (3), e000060.
24. Sebens, S.; Schafer, H., The tumor stroma as mediator of drug resistance--a potential target to improve cancer therapy? *Current pharmaceutical biotechnology* **2012,** *13* (11), 2259-72.
25. Campos-Parra, A. D.; Zuloaga, C.; Manriquez, M. E.; Aviles, A.; Borbolla-Escoboza, J.; Cardona, A.; Meneses, A.; Arrieta, O., KRAS mutation as the biomarker of response to chemotherapy and EGFR-TKIs in patients with advanced non-small cell lung cancer: clues for its potential use in second-line therapy decision making. *American journal of clinical oncology* **2015,** *38* (1), 33-40.
26. Zhang, J.; Babic, A., Regulation of the MET oncogene: molecular mechanisms. *Carcinogenesis* **2016,** *37* (4), 345-355.
27. Yun, H. S.; Baek, J. H.; Yim, J. H.; Um, H. D.; Park, J. K.; Song, J. Y.; Park, I. C.; Kim, J. S.; Lee, S. J.; Lee, C. W.; Hwang, S. G., Radiotherapy diagnostic biomarkers in radioresistant human H460 lung cancer stem-like cells. *Cancer biology & therapy* **2016,** *17* (2), 208-18.
28. Haffner, C.; Frauli, M.; Topp, S.; Irmler, M.; Hofmann, K.; Regula, J. T.; Bally-Cuif, L.; Haass, C., Nicalin and its binding partner Nomo are novel Nodal signaling antagonists. *The EMBO journal* **2004,** *23* (15), 3041-50.

29. Kalyan, A.; Carneiro, B. A.; Chandra, S.; Kaplan, J.; Chae, Y. K.; Matsangou, M.; Hendrix, M. J. C.; Giles, F., Nodal Signaling as a Developmental Therapeutics Target in Oncology. *Molecular cancer therapeutics* **2017,** *16* (5), 787-792.
30. He, P.; Wu, W.; Wang, H.; Liao, K.; Zhang, W.; Xiong, G.; Wu, F.; Meng, G.; Yang, K., Co-expression of Rho guanine nucleotide exchange factor 5 and Src associates with poor prognosis of patients with resected non-small cell lung cancer. *Oncology reports* **2013,** *30* (6), 2864-70.
31. Komiya, Y.; Onodera, Y.; Kuroiwa, M.; Nomimura, S.; Kubo, Y.; Nam, J. M.; Kajiwara, K.; Nada, S.; Oneyama, C.; Sabe, H.; Okada, M., The Rho guanine nucleotide exchange factor ARHGEF5 promotes tumor malignancy via epithelial-mesenchymal transition. *Oncogenesis* **2016,** *5* (9), e258.
32. DiBardino, D. M.; Rawson, D. W.; Saqi, A.; Heymann, J. J.; Pagan, C. A.; Bulman, W. A., Next-generation sequencing of non-small cell lung cancer using a customized, targeted sequencing panel: Emphasis on small biopsy and cytology. *CytoJournal* **2017,** *14*, 7.
33. Gadea, G.; Blangy, A., Dock-family exchange factors in cell migration and disease. *European journal of cell biology* **2014,** *93* (10-12), 466-77.
34. Gordon, E. A.; Whisenant, T. C.; Zeller, M.; Kaake, R. M.; Gordon, W. M.; Krotee, P.; Patel, V.; Huang, L.; Baldi, P.; Bardwell, L., Combining docking site and phosphosite predictions to find new substrates: identification of smoothelin-like-2 (SMTNL2) as a c-Jun N-terminal kinase (JNK) substrate. *Cellular signalling* **2013,** *25* (12), 2518-29.
35. Kim, B. H.; Shenoy, A. R.; Kumar, P.; Das, R.; Tiwari, S.; MacMicking, J. D., A family of IFN-gamma-inducible 65-kD GTPases protects against bacterial infection. *Science (New York, N.Y.)* **2011,** *332* (6030), 717-21.
36. Miura, K., ERK2-binding domain is required for phosphorylation of EBITEIN1, a potential downstream interactor of ERK2. *Biochemical and biophysical research communications* **2008,** *375* (3), 367-71.
37. Silva, M. P.; Barros-Silva, J. D.; Vieira, J.; Lisboa, S.; Torres, L.; Correia, C.; Vieira-Coimbra, M.; Martins, A. T.; Jeronimo, C.; Henrique, R.; Paulo, P.; Teixeira, M. R., NCOA2 is a candidate target gene of 8q gain associated with clinically aggressive prostate cancer. *Genes, chromosomes & cancer* **2016,** *55* (4), 365-74.
38. Xiao, Z. G.; Shen, J.; Zhang, L.; Li, L. F.; Li, M. X.; Hu, W.; Li, Z. J.; Cho, C. H., The Roles of Histone Demethylase UTX and JMJD3 (KDM6B) in Cancers: Current Progress and Future Perspectives. *Current medicinal chemistry* **2016,** *23* (32), 3687-3696.
39. Xu, G.; Zhang, M.; Zhu, H.; Xu, J., A 15-gene signature for prediction of colon cancer recurrence and prognosis based on SVM. *Gene* **2017,** *604*, 33-40.
40. Ali, G.; Bruno, R.; Poma, A. M.; Affinito, O.; Monticelli, A.; Piaggi, P.; Ricciardi, S.; Lucchi, M.; Melfi, F.; Chella, A.; Cocozza, S.; Fontanini, G., Whole transcriptome targeted gene quantification provides new insights on pulmonary sarcomatoid carcinomas. *Scientific reports* **2019,** *9* (1), 3536.
41. Cathcart-Rake, E.; Lopez-Chavez, A., Young male with fanconi anemia and EGFR-mutant non-small-cell lung cancer. *Journal of thoracic oncology : official publication of the International Association for the Study of Lung Cancer* **2014,** *9* (11), e83-5.
42. Pfaffle, H. N.; Wang, M.; Gheorghiu, L.; Ferraiolo, N.; Greninger, P.; Borgmann, K.; Settleman, J.; Benes, C. H.; Sequist, L. V.; Zou, L.; Willers, H., EGFR-activating mutations correlate with a Fanconi anemia-like cellular phenotype that includes PARP inhibitor sensitivity. *Cancer research* **2013,** *73* (20), 6254-63.
43. Ye, H.; Zhang, X.; Chen, Y.; Liu, Q.; Wei, J., Ranking novel cancer driving synthetic lethal gene pairs using TCGA data. *Oncotarget* **2016,** *7* (34), 55352-55367.

44. Garcia-Higuera, I.; Taniguchi, T.; Ganesan, S.; Meyn, M. S.; Timmers, C.; Hejna, J.; Grompe, M.; D'Andrea, A. D., Interaction of the Fanconi anemia proteins and BRCA1 in a common pathway. *Molecular cell* **2001,** *7* (2), 249-62.

45. Li, L.; Gu, X.; Yue, J.; Zhao, Q.; Lv, D.; Chen, H.; Xu, L., Acquisition of EGFR TKI resistance and EMT phenotype is linked with activation of IGF1R/NF-kappaB pathway in EGFR-mutant NSCLC. *Oncotarget* **2017,** *8* (54), 92240-92253.

46. Matusan-Ilijas, K.; Damante, G.; Fabbro, D.; Dordevic, G.; Hadzisejdic, I.; Grahovac, M.; Avirovic, M.; Grahovac, B.; Jonjic, N.; Lucin, K., EGFR expression is linked to osteopontin and Nf-kappaB signaling in clear cell renal cell carcinoma. *Clinical & translational oncology : official publication of the Federation of Spanish Oncology Societies and of the National Cancer Institute of Mexico* **2013,** *15* (1), 65-71.

47. Meylan, E.; Dooley, A. L.; Feldser, D. M.; Shen, L.; Turk, E.; Ouyang, C.; Jacks, T., Requirement for NF-kappaB signalling in a mouse model of lung adenocarcinoma. *Nature* **2009,** *462* (7269), 104-7.

48. Baud, V.; Collares, D., Post-Translational Modifications of RelB NF-kappaB Subunit and Associated Functions. *Cells* **2016,** *5* (2).

49. Lin, C.; Lu, W.; Ren, Z.; Tang, Y.; Zhang, C.; Yang, R.; Chen, Y.; Cao, W.; Wang, L.; Wang, X.; Ji, T., Elevated RET expression enhances EGFR activation and mediates EGFR inhibitor resistance in head and neck squamous cell carcinoma. *Cancer letters* **2016,** *377* (1), 1-10.

50. Wang, S. E.; Narasanna, A.; Perez-Torres, M.; Xiang, B.; Wu, F. Y.; Yang, S.; Carpenter, G.; Gazdar, A. F.; Muthuswamy, S. K.; Arteaga, C. L., HER2 kinase domain mutation results in constitutive phosphorylation and activation of HER2 and EGFR and resistance to EGFR tyrosine kinase inhibitors. *Cancer cell* **2006,** *10* (1), 25-38.

51. Guo, G.; Gong, K.; Wohlfeld, B.; Hatanpaa, K. J.; Zhao, D.; Habib, A. A., Ligand-Independent EGFR Signaling. *Cancer research* **2015,** *75* (17), 3436-41.

52. Han, W.; Carpenter, R. L.; Cao, X.; Lo, H. W., STAT1 gene expression is enhanced by nuclear EGFR and HER2 via cooperation with STAT3. *Molecular carcinogenesis* **2013,** *52* (12), 959-69.

53. Byers, L. A.; Diao, L.; Wang, J.; Saintigny, P.; Girard, L.; Peyton, M.; Shen, L.; Fan, Y.; Giri, U.; Tumula, P. K.; Nilsson, M. B.; Gudikote, J.; Tran, H.; Cardnell, R. J.; Bearss, D. J.; Warner, S. L.; Foulks, J. M.; Kanner, S. B.; Gandhi, V.; Krett, N.; Rosen, S. T.; Kim, E. S.; Herbst, R. S.; Blumenschein, G. R.; Lee, J. J.; Lippman, S. M.; Ang, K. K.; Mills, G. B.; Hong, W. K.; Weinstein, J. N.; Wistuba, II; Coombes, K. R.; Minna, J. D.; Heymach, J. V., An epithelial-mesenchymal transition gene signature predicts resistance to EGFR and PI3K inhibitors and identifies Axl as a therapeutic target for overcoming EGFR inhibitor resistance. *Clinical cancer research : an official journal of the American Association for Cancer Research* **2013,** *19* (1), 279-90.

54. Elkabets, M.; Pazarentzos, E.; Juric, D.; Sheng, Q.; Pelossof, R. A.; Brook, S.; Benzaken, A. O.; Rodon, J.; Morse, N.; Yan, J. J.; Liu, M.; Das, R.; Chen, Y.; Tam, A.; Wang, H.; Liang, J.; Gurski, J. M.; Kerr, D. A.; Rosell, R.; Teixido, C.; Huang, A.; Ghossein, R. A.; Rosen, N.; Bivona, T. G.; Scaltriti, M.; Baselga, J., AXL mediates resistance to PI3Kalpha inhibition by activating the EGFR/PKC/mTOR axis in head and neck and esophageal squamous cell carcinomas. *Cancer cell* **2015,** *27* (4), 533-46.

55. Ghiso, E.; Migliore, C.; Ciciriello, V.; Morando, E.; Petrelli, A.; Corso, S.; De Luca, E.; Gatti, G.; Volante, M.; Giordano, S., YAP-Dependent AXL Overexpression Mediates Resistance to EGFR Inhibitors in NSCLC. *Neoplasia (New York, N.Y.)* **2017,** *19* (12), 1012-1021.

56. Guo, G.; Gong, K.; Ali, S.; Ali, N.; Shallwani, S.; Hatanpaa, K. J.; Pan, E.; Mickey, B.; Burma, S.; Wang, D. H.; Kesari, S.; Sarkaria, J. N.; Zhao, D.; Habib, A. A., A TNF-JNK-Axl-ERK signaling axis mediates primary resistance to EGFR inhibition in glioblastoma. *Nature neuroscience* **2017,** *20* (8), 1074-1084.

57. Tian, Y.; Zhang, Z.; Miao, L.; Yang, Z.; Yang, J.; Wang, Y.; Qian, D.; Cai, H.; Wang, Y., Anexelekto (AXL) Increases Resistance to EGFR-TKI and Activation of AKT and ERK1/2 in Non-Small Cell Lung Cancer Cells. *Oncology research* **2016,** *24* (5), 295-303.

58. Qu, Y.; Wu, X.; Yin, Y.; Yang, Y.; Ma, D.; Li, H., Antitumor activity of selective MEK1/2 inhibitor AZD6244 in combination with PI3K/mTOR inhibitor BEZ235 in gefitinib-resistant NSCLC xenograft models. *Journal of experimental & clinical cancer research : CR* **2014,** *33*, 52.

59. Buonato, J. M.; Lazzara, M. J., ERK1/2 blockade prevents epithelial-mesenchymal transition in lung cancer cells and promotes their sensitivity to EGFR inhibition. *Cancer research* **2014,** *74* (1), 309-19.

60. Schmitt, N. C.; Trivedi, S.; Ferris, R. L., STAT1 Activation Is Enhanced by Cisplatin and Variably Affected by EGFR Inhibition in HNSCC Cells. *Molecular cancer therapeutics* **2015,** *14* (9), 2103-11.

61. Cheng, C. C.; Lin, H. C.; Tsai, K. J.; Chiang, Y. W.; Lim, K. H.; Chen, C. G.; Su, Y. W.; Peng, C. L.; Ho, A. S.; Huang, L.; Chang, Y. C.; Lin, H. C.; Chang, J.; Chang, Y. F., Epidermal growth factor induces STAT1 expression to exacerbate the IFNr-mediated PD-L1 axis in epidermal growth factor receptor-positive cancers. *Molecular carcinogenesis* **2018,** *57* (11), 1588-1598.

62. Phuchareon, J.; McCormick, F.; Eisele, D. W.; Tetsu, O., EGFR inhibition evokes innate drug resistance in lung cancer cells by preventing Akt activity and thus inactivating Ets-1 function. *Proceedings of the National Academy of Sciences of the United States of America* **2015,** *112* (29), E3855-63.

63. Tetsu, O.; Eisele, D. W.; McCormick, F., Resistance to EGFR-targeted therapy by Ets-1 inactivation. *Cell cycle (Georgetown, Tex.)* **2015,** *14* (20), 3211-3212.

© 2019 by the authors. Submitted for possible open access publication under the terms and conditions of the Creative Commons Attribution (CC BY) license (http://creativecommons.org/licenses/by/4.0/).
